# Improvement in gait velocity variability after cerebrospinal fluid elimination and its relationship to clinical symptoms in patients with idiopathic normal pressure hydrocephalus

**DOI:** 10.1111/ggi.14915

**Published:** 2024-05-29

**Authors:** Takahiro Yamamoto, Ryoko Fujito, Yoshihiro Chadani, Tetsuo Kashibayashi, Naoto Kamimura, Atsushi Tsuda, Masanori Akamatsu, Takuya Matsushita, Takuji Yamagami, Tetsuya Ueba, Motoaki Saito, Keiji Inoue, Masashi Izumi, Hiroaki Kazui

**Affiliations:** ^1^ Department of Neuropsychiatry, Kochi Medical School Kochi University Nankoku Kochi Japan; ^2^ Department of Rehabilitation Center Kochi Medical School Hospital Nankoku Kochi Japan; ^3^ Department of Neuropsychiatry Hyogo Prefectural Rehabilitation Hospital at Nishi‐Harima Tatsuno Hyogo Japan; ^4^ Health Service Center Medical School Branch Kochi University Nankoku Kochi Japan; ^5^ Department of Neurology, Kochi Medical School Kochi University Nankoku Kochi Japan; ^6^ Department of Diagnostic and Interventional Radiology, Kochi Medical School Kochi University Nankoku Kochi Japan; ^7^ Department of Neurosurgery, Kochi Medical School Kochi University Nankoku Kochi Japan; ^8^ Department of Pharmacology, Kochi Medical School Kochi University Nankoku Kochi Japan; ^9^ Department of Urology, Kochi Medical School Kochi University Nankoku Kochi Japan; ^10^ Department of Orthopaedic Surgery, Kochi Medical School Kochi University Nankoku Kochi Japan

**Keywords:** cerebrospinal fluid tap test, cognitive impairment, gait velocity variability, idiopathic normal pressure hydrocephalus, Timed Up and Go Test

## Abstract

**Aim:**

This study aimed to investigate the improvement in gait velocity variability after cerebrospinal fluid (CSF) elimination, and the association between gait velocity variability and gait and cognitive impairment in patients with idiopathic normal pressure hydrocephalus.

**Methods:**

The gait velocity of 44 patients with idiopathic normal pressure hydrocephalus was measured using the Timed Up and Go Test (TUG) for a total of 10 times over 3 days each before and after CSF elimination. The coefficient of variation (CV) in the time required for the sequence of actions in TUG (TUG‐CV) was calculated using 10 TUG data, and used for measuring intraindividual gait velocity variability. Gait quality was evaluated with the Gait Status Scale Revised (GSSR), and cognitive function was evaluated with the Mini‐Mental State Examination and the Frontal Assessment Battery.

**Results:**

The TUG, TUG‐CV, GSSR and Frontal Assessment Battery results improved significantly after CSF elimination. The analyses using pre‐CSF elimination results showed that the TUG‐CV significantly and positively correlated with the TUG and GSSR results, and negatively with Mini‐Mental State Examination results, but not with age and the Frontal Assessment Battery results. The stepwise multiple regression analysis indicates that the TUG, GSSR and Mini‐Mental State Examination results were significant predictors of the TUG‐CV. The analysis using data of change after CSF elimination showed that ΔTUG and ΔGSSR were significant predictors of ΔTUG‐CV.

**Conclusions:**

Gait velocity variability improved after CSF elimination, and gait velocity variability was associated with gait disturbances and cognitive impairment in patients with idiopathic normal pressure hydrocephalus. **Geriatr Gerontol Int 2024; 24: 693–699**.

## Graphical abstract

The time required for the sequence of actions in the Timed Up and Go Test was measured 10 times each before and after cerebrospinal fluid elimination, and intraindividual gait velocity variability was evaluated in 44 patients with idiopathic normal pressure hydrocephalus. We found that gait velocity variability was associated with gait and cognitive impairment, and that it improved after cerebrospinal fluid elimination.

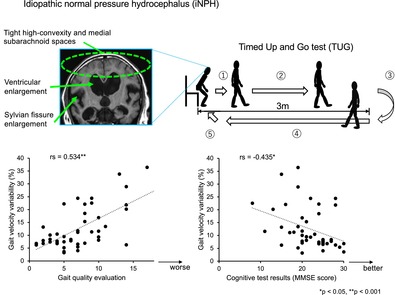

## Introduction

Normal pressure hydrocephalus (NPH) is a condition that presents with gait disturbance, cognitive impairment and urinary incontinence (triad symptoms) in patients with enlarged ventricles under normal cerebrospinal fluid (CSF) pressure, and the triad symptoms can be treated by shunt surgery.^1^, ^2^ Idiopathic NPH (iNPH), without antecedent diseases, is a common condition with a prevalence of the weighted average of 1.6% in four population‐based studies in Japan^3^, ^4^, ^5^, ^6^ and 3.7% in a study in Sweden.^7^ Three large studies have investigated the effect of shunt surgery on iNPH.^8^, ^9^, ^10^ These studies have shown similar results, where 60%–70% of patients with iNPH showed a remarkable improvement in their activities of daily living 1 year after shunt surgery.

Gait disturbance is the most prominent clinical symptom of iNPH.^11^ In patients with iNPH, gait disturbance includes various features, such as small‐step gait, magnet gait, broad‐based gait, festinating gait, freezing of gait, lateral sway and outward walking, and gait velocity slows down.^12^, ^13^, ^14^ Recently, attention has focused on gait variability in iNPH. Patients with iNPH have shown increased variability in the time required for one step, and this variability has decreased after CSF elimination in patients with iNPH.^13^, ^15^ In addition, falls are important clinical symptoms of iNPH, because they can result in injury, loss of independence and decreased life expectancy.^16^, ^17^, ^18^ Variability in the time required for one step is a useful indicator of falls in iNPH.^19^ In Parkinson's disease, where gait disturbance resembles iNPH, gait speed fluctuations are a risk factor for falls.^20^ Furthermore, falls in older adults are not correlated with gait velocity, but are significantly correlated with variability in the time required for one step.^21^, ^22^ However, evaluations of gait variability in the previous studies were based on data obtained from a single measurement^15^ or data measured simultaneously on the same day, although data were measured multiple times.^13^, ^23^

In the clinical settings of patients with iNPH, the CSF tap test is widely used for diagnosing iNPH and predicting the therapeutic effect of shunt surgery.^11^, ^24^ In the CSF tap test, improvement of gait disturbances after CSF elimination is the easiest to evaluate and most reliable. The Timed Up and Go Test (TUG)^25^ measures in seconds the time taken by an individual to stand up from a standard armchair, walk a distance of 3 m, turn, walk back to the chair and sit down again. A significant improvement after CSF elimination in TUG is defined as a decrease in time of ≥10%.^24^ However, in accurately determining the presence or absence of improvement after CSF elimination, knowing whether gait velocity fluctuates in patients with iNPH and what influences gait velocity variability is necessary.

In the present study, TUG was carried out a total of 10 times over 3 days each before and after CSF elimination, and the gait velocity variability of patients with iNPH was measured. Then, gait velocity variability was compared before and after CSF elimination, and the association between gait velocity variability and gait and cognitive impairments in patients with iNPH was evaluated.

## Methods

The present retrospective study was approved by the ethics committee of the Kochi University Medical Hospital (Kochi, Japan), and followed the Guidelines for Good Clinical Practice and the Declaration of Helsinki. As this was a retrospective observational study, in which data were collected from medical records, an opt‐out approach was used; that is, information regarding the study was provided on our homepage, and each participant's consent to take part in the study was considered to be obtained unless they expressed their desire to be excluded.

### Participants

The inclusion criteria of the participants were as follows: (i) patients who were hospitalized in the Department of Neuropsychiatry Kochi University Medical Hospital between March 2018 and January 2023, (ii) those who underwent a CSF tap test, and (iii) those who met the diagnostic criteria for probable iNPH of the Japanese clinical guidelines for iNPH second edition.^24^ The criteria for probable iNPH were as follows: (i) onset age >60 years; (ii) presence of at least one of the iNPH triad of symptoms; (iii) presence of ventricular enlargement on magnetic resonance images (Evans index >0.3); (iv) absence of other diseases, conditions or radiological findings that could be causing the clinical manifestations; (v) no history or evidence of underlying diseases that can cause secondary NPH; (vi) normal CSF pressure (i.e. ≤20 cm H_2_O) and contents; and (vii) one of the following: presence of neuroimaging feature termed “disproportionately enlarged subarachnoid space hydrocephalus (DESH)” under the presence of gait disturbance, or improvement in one or more of the triad symptoms after removing 30 mL of CSF via lumbar tap (CSF tap test). The DESH refers to the “narrowing of the sulci and subarachnoid spaces over the high convexity/midline surface with ventricular enlargement.” Improvement of symptoms after the lumbar tap was defined as an improvement of ≥3 points in the Mini‐Mental State Examination (MMSE)^26^ or an improvement of at least 10% of the time required for a series of movements in TUG.

### Assessment

Time in seconds required to carry out a sequence of actions in TUG was used as an index of gait velocity. Gait quality was evaluated with the Gait Status Scale Revised (GSSR).^27^ The GSSR can be used to evaluate several aspects of gait disturbance in iNPH^28^, ^29^ and measure its improvement after shunt surgery.^27^, ^30^ The scale focuses on 10 factors related to gait disturbances in iNPH: postural stability, walking independence, wide base gait, lateral sway, petit pas gait, festinating gait, freezing of gait, disturbed tandem walking, shuffle, and bow‐leggedness. The total GSSR score was calculated from the 10‐item scores, and it was distributed between 0 and 18. The intraclass correlation coefficients (ICCs) for the total GSSR score were calculated, with 0.985 being sufficiently high. The ICCs were calculated using 12 total GSSR scores obtained from two raters who independently evaluated the gait quality of eight patients with iNPH who were not included in this study. Six raters (physiotherapists, geriatric psychiatrists, or residents) participated in the gait quality assessment to calculate the ICCs. Overall cognitive function was evaluated with the MMSE, and frontal lobe function was evaluated with the Frontal Assessment Battery (FAB).^31^ The severity of the triad symptoms of iNPH was evaluated with the iNPH grading scale (iNPHGS).^12^ The score for each symptom ranged from 0 to 4. Social disadvantage and behavioral limitations were evaluated with the modified Rankin scale (mRS).^32^ The mRS ranged from grades 0 to 5. Higher scores indicated better performance in MMSE and FAB, and worse performance in TUG, GSSR, iNPHGS and mRS.

### Procedure

At Kochi University Medical Hospital, specialists in geriatric psychiatry, neurology, rehabilitation medicine, neuro‐urology, and neuroradiology attend to the clinical and neuroimaging examinations of patients who meet the diagnostic criteria for possible iNPH, and neurosurgeons perform shunt surgery on patients deemed eligible.^29^ The diagnostic criteria and treatment procedures for patients with iNPH followed the second^24^ or third^11^ editions of the Japanese clinical guidelines for iNPH. Preshunt surgery examinations, including the CSF tap test, were primarily carried out in the psychiatry department.

**Figure 1:**
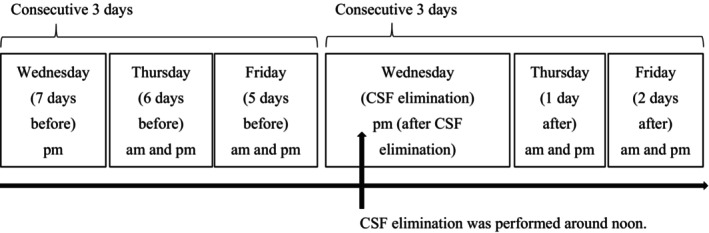
Repeated Timed Up and Go Test (TUG) measurement schedule before and after cerebrospinal fluid (CSF) elimination. TUG was carried out twice in one trial. The trial was repeated five times each before and after CSF elimination. CSF elimination was carried out on 1 day. Up to 30 mL of CSF was removed by lumbar puncture. The CSF was removed until the CSF pressure reached 0 mmH_2_O when the amount removed did not reach 30 mL. Before CSF elimination, TUG trials were carried out in the afternoon 7 days in the morning, and afternoon 6 and 5 days before the day of CSF elimination. After CSF elimination, TUG trials were carried out in the afternoon on the day of CSF elimination, and in the morning and afternoon after 1 and 2 days after the day of CSF elimination. Thus, the 10 TUG data were obtained each before and after CSF elimination in one patient with idiopathic normal pressure hydrocephalus. The days for evaluating TUG were fixed to Wednesday, Thursday and Friday to exclude Saturday and Sunday holidays from the three consecutive days. Mondays are also excluded because of the many holidays in Japan.

The schedule of repeated TUG in our psychiatry department is shown in Fig. 1. Intraindividual variability of time in seconds required for the sequence of actions in TUG was expressed in terms of coefficient of variation (CV) calculated by the following equation: CV (%) = standard deviation / mean × 100.^33^ The CV in TUG (TUG‐CV) was calculated before and after CSF elimination using the 10 TUG data each before and after CSF elimination. Evaluations with the GSSR, MMSE, FAB, iNPHGS and mRS were carried out between 1 week and 1 day before CSF elimination, the GSSR was evaluated 1 day after, and other evaluations were carried out 1 week after CSF elimination.

### Statistical analysis

When comparing TUG data before and after CSF elimination and evaluating the relationship between TUG data and other test results, the average of the two TUG data of the first trial was used both before and after CSF elimination. The results of the TUG‐CV, TUG, GSSR, MMSE, FAB, gait, cognitive, and urinary domains of iNPHGS and mRS were compared before and after CSF elimination. The normality of the distribution of all variables was tested with the Shapiro–Wilk test. For normally distributed variables, the paired *t*‐test was used, and for non‐normally distributed variables, the Wilcoxon signed‐rank test was used. Correlations between the TUG‐CV and other evaluations were assessed using Spearman's rank correlation coefficient. These analyses were carried out using the evaluation results before CSF elimination, and changes in the results after CSF elimination. In addition, stepwise multiple regression analysis was carried out using TUG‐CV before CSF elimination as the objective variable, and TUG, GSSR, MMSE and FAB results before CSF elimination, and age as explanatory variables. This analysis was also carried out using changes in the results after CSF elimination. Before carrying out multiple regression analyses, multivariate normality between the variables was checked by a P–P plot. Statistical analyses were carried out using IBM SPSS version 26 (IBM Corporation, Armonk, NY, USA). All statistical significance was set at the level of *P* < 0.05.

## Results

Data of 44 patients with iNPH (20 men and 24 women) were analyzed in the present study. The mean age of the participants was 77.4 ± 5.3 years (range 63–91 years).

Of the 44 patients, before the CSF elimination, eight of 10 TUG data were obtained for one patient, and after the CSF elimination, eight TUG data were obtained for two patients and six TUG data for one patient. For these four patients, TUG‐CV was calculated with the data that could be collected. The TUG, TUG‐CV, GSSR, FAB, and gait and urinary domains of iNPHGS and mRS results improved significantly after CSF elimination (Table 1). A total of 15 raters (physiotherapists, geriatric psychiatrists or residents) participated in the GSSR evaluation of the 44 patients with iNPH in this study.

**Table 1:** Comparison of evaluations of patients with idiopathic normal pressure hydrocephalus before and after cerebrospinal fluid elimination

Evaluations	Before CSF elimination	After CSF elimination	*P*‐value
TUG (s)	21.4 ± 19.1	17.5 ± 12.2	<0.001^†^
TUG‐CV	12.7 ± 8.2	8.7 ± 4.1	0.007^†^
GSSR	7.1 ± 3.9	5.8 ± 3.7	<0.001^†^
MMSE	21.7 ± 5.1	22.4 ± 5.1	0.279^†^
FAB	10.8 ± 3.1	11.5 ± 3.2	0.007^‡^
iNPHGS score			
Gait	1.9 ± 0.6	1.7 ± 0.8	0.005^†^
Cognition	2.2 ± 0.9	2.1 ± 1.0	0.248^†^
Urination	1.7 ± 1.2	1.4 ± 1.1	0.003^†^
mRS	2.3 ± 0.9	2.2 ± 0.8	0.046^†^

Mean ± standard deviation.

^†^ Wilcoxon signed‐rank sum test.

^‡^ Paired *t*‐test.

CSF, cerebrospinal fluid; FAB, Frontal Assessment Battery; GSSR, Gait Status Scale Revised; iNPHGS, idiopathic normal pressure hydrocephalus grading scale; MMSE, Mini‐Mental State Examination; mRS, modified Rankin scale; TUG, Timed Up and Go Test; TUG‐CV, coefficient of variation in the time required for the sequence of actions in the Timed Up and Go Test, which was calculated using 10 Timed Up and Go Test data.

In the pre‐CSF elimination data, the TUG‐CV significantly correlated with the TUG (rs = 0.677, *P* < 0.001), GSSR (rs = 0.534, *P* < 0.001), MMSE (rs = −0.435, *P* = 0.003), and iNPHGS gait (rs = 0.564, *P* < 0.001), cognition (rs = 0.368, *P* = 0.014) and urination (rs = 0.366, *P* = 0.014) domains, and mRS (rs = 0.502, *P* = 0.001). However, TUG‐CV was not significantly correlated with age (rs = −0.033, *P* = 0.831) or FAB (rs = −0.266, *P* = 0.081).

The change in the TUG‐CV after CSF elimination (ΔTUG‐CV) significantly correlated with the change in the GSSR after CSF elimination (ΔGSSR; rs = 0.476, *P* = 0.001). However, ΔTUG‐CV was not significantly correlated with age (rs = −0.143, *P* = 0.354) or the changes in the TUG (ΔTUG; rs = 0.277, *P* = 0.072), MMSE (ΔMMSE; rs = −0.113, *P* = 0.465) or FAB (ΔFAB; rs = −0.112, *P* = 0.468) after CSF elimination.

**Figure 2:**
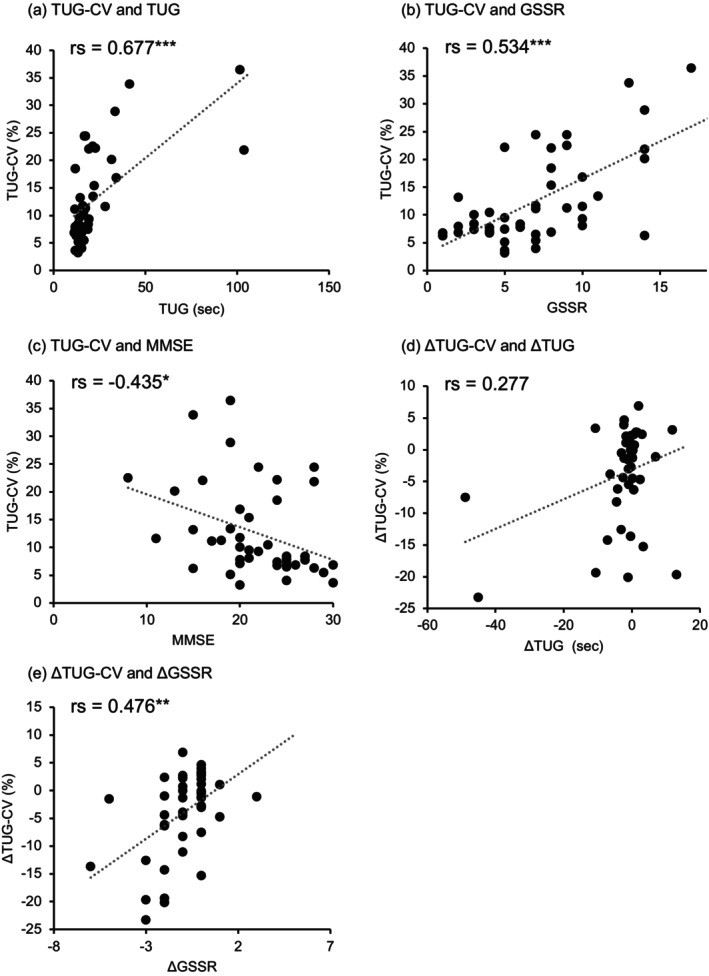
Scatterplots of (a) the coefficient of variation in the time required for the sequence of actions in the Timed Up and Go Test (TUG‐CV) and Timed Up and Go Test (TUG), (b) TUG‐CV and Gait Status Scale Revised (GSSR), (c) TUG‐CV and Mini‐Mental State Examination (MMSE), (d) ΔTUG‐CV and ΔTUG, and (e) ΔTUG‐CV and ΔGSSR. The (a) TUG, (b) GSSR and (c) MMSE were significant predictors of TUG‐CV. (d) The ΔTUG and (e) ΔGSSR were significant predictors of ΔTUG‐CV. *P < 0.05, **P < 0.01 and ***P < 0.001.

The normality of distributions of the objective and explanatory variables before CSF elimination, and the normality of distributions of the changes in the variables after CSF elimination were confirmed. No multicollinearity was observed. The stepwise multiple regression analysis using data before CSF tapping showed that the TUG, GSSR and MMSE were significant predictors of the TUG‐CV (Table 2 and Fig. 2). A similar analysis using data changes after CSF tapping showed that the ΔTUG and ΔGSSR were significant predictors of the ΔTUG‐CV (Table 3 and Fig. 2).

**Table 2:** Results of stepwise multiple regression analysis to predict coefficient of variation in the time required for the sequence of actions in the Timed Up and Go Test before cerebrospinal fluid elimination using variables before cerebrospinal fluid elimination

Variables	B	SE	Beta	*P*‐value	95% CI
TUG	0.178	0.059	0.416	0.005	0.058, 0.298
GSSR	0.646	0.3	0.307	0.038	0.039, 1.252
MMSE	−0.405	0.178	−0.25	0.029	−0.765, −0.044

The analysis was carried out using coefficient of variation in the time required for the sequence of actions in the Timed Up and Go Test before cerebrospinal fluid elimination as the objective variable, and Timed Up and Go Test, Gait Status Scale Revised, Mini‐Mental State Examination and Frontal Assessment Battery before cerebrospinal fluid elimination, and age as explanatory variables. The results showed that the Timed Up and Go Test, Gait Status Scale Revised and Mini‐Mental State Examination were significant predictors of coefficient of variation in the time required for the sequence of actions in the Timed Up and Go Test.

B, partial regression coefficient; CSF, cerebrospinal fluid; GSSR, Gait Status Scale Revised; MMSE, Mini‐Mental State Examination; SE, standard error; TUG, Timed Up and Go Test; TUG‐CV, coefficient of variation in the time required for the sequence of actions in the Timed Up and Go Test.

**Table 3:** Results of stepwise multiple regression analysis to predict change in the coefficient of variation in the time required for the sequence of actions in the Timed Up and Go Test after cerebrospinal fluid elimination using changes in variables after cerebrospinal fluid elimination

Variables	B	SE	Beta	p value	95% CI
ΔTUG	0.198	0.093	0.288	0.04	0.010, 0.387
ΔGSSR	2.176	0.652	0.451	0.02	0.855, 3.496

The analysis was carried out using change in coefficient of variation in the time required for the sequence of actions in the Timed Up and Go Test after cerebrospinal fluid elimination as the objective variable, and change in Timed Up and Go Test, change in Gait Status Scale Revised, change in Mini‐Mental State Examination and change in Frontal Assessment Battery after cerebrospinal fluid elimination, and age as explanatory variables. The results showed that change in Timed Up and Go Test and change in Gait Status Scale Revised were significant predictors of change in coefficient of variation in the time required for the sequence of actions in the Timed Up and Go Test.

B, partial regression coefficient; CSF, cerebrospinal fluid; GSSR, Gait Status Scale Revised; SE, standard error; TUG‐CV, coefficient of variation in the time required for the sequence of actions in the Timed Up and Go Test.

## Discussion

In the present study, TUG was carried out 10 times over 3 days each before and after CSF elimination on nearly all 44 patients with iNPH. Then, the TUG‐CV was calculated from the 10 TUG data each before and after CSF elimination. The TUG‐CV significantly decreased after CSF elimination, indicating that the gait velocity variability might be one of the gait symptoms in patients with iNPH. In a previous study in which patients with iNPH were instructed to walk 10 m at a self‐selected speed in a single session that consisted of at least 10 trials, gait repeatability was evaluated by the ICC.^23^ In the study, the ICCs of velocity, step length, cadence and step time were 0.94, 0.95, 0.79 and 0.78, respectively. The study concluded good gait repeatability in patients with iNPH, which means that gait variability was low. However, ICCs were not evaluated after CSF elimination, so ICCs could improve after CSF elimination in that study.

In the other study, patients with iNPH were instructed to walk 5.8 m four times, and the trial was carried out once before and once after CSF elimination.^13^ The variabilities in step length and step time increased in patients with iNPH compared with those in control participants, and the variabilities in patients with iNPH improved after CSF elimination. In another study, patients with iNPH were instructed to walk 15 m twice a day, and the trial was carried out once before and once after CSF elimination.^15^ The variabilities in step time and movement trajectory amplitude of the center of the body in both medial/lateral and vertical directions increased in patients with iNPH compared with those in the control participants, and the variabilities in patients with iNPH decreased after CSF elimination.

In these previous studies, the variability over days was not evaluated. Thus, we were the first to show that in patients with iNPH, gait variability across days improves after CSF elimination.

Gait velocity variability significantly correlated with TUG, GSSR and iNPHGS gait scores, and the degree of improvement of gait velocity variability after CSF elimination significantly correlated with the improvement in the GSSR score after CSF elimination in the present study. In this study, stepwise multiple regression analyses showed that TUG and GSSR scores were significant predictors of gait velocity variability, and that the improvement in GSSR scores after CSF elimination was also a significant predictor of the degree of improvement in gait velocity variability after CSF elimination. These results might indicate that the various gait disturbances that occur in iNPH contribute most to the gait velocity variability in patients with iNPH. GSSR includes items to assess characteristic gait symptoms observed in patients with iNPH, such as small step length, freezing of gait, festinating gait, lateral sway and postural stability. Variabilities in step length and step time increased in iNPH.^13^ This might be the cause of gait velocity variability.

In the analysis using data before CSF elimination in the present study, the TUG‐CV significantly correlated with the MMSE and iNPHGS cognition scores, but not with the FAB score. In addition, the stepwise multiple regression analysis showed that the MMSE score, but not the FAB score, was a significant predictor of the TUG‐CV in this study. TUG requires memorizing the series of movements. The TUG also requires an executive function to complete the complex movement procedures. In fact, memory and executive functions are related to TUG performance.^34^

In a previous study, the variabilities of step length and step time in patients with iNPH correlated with the FAB score,^13^ which might appear to be inconsistent with the results of the present study. However, in a previous study, the association between the variability of gait disturbances and the MMSE score was not evaluated. Thus, the significant correlation found with the FAB score in the previous study was not specific to frontal lobe function, but might be a manifestation of correlation with cognitive function. This assumption might be supported by the finding that patients with more severe cognitive impairment showed greater variability in TUG performance.^35^

The findings of the present study showed that CSF elimination could reduce the variability in gait velocity. Variability in the time required for one step is associated with falls in iNPH.^19^ Thus, shunt surgery could reduce falls in patients with iNPH. In the CSF tap test, whether gait velocity improves after CSF elimination is the most commonly assessed outcome. Therefore, variability in gait velocity might cause false positives and negatives in the test. This study showed that patients with slow gait, many features of gait disturbance and pronounced cognitive impairment had high variability in gait velocity. Therefore, whether CSF elimination improves symptoms in such patients should be carefully determined. Multiple measurements before and after CSF elimination, and the use of the fastest or average values might improve the determination accuracy.

Here, intraindividual gait velocity variability was assessed using TUG‐CV. A previous study used CV to measure intraindividual gait velocity variability.^33^ In addition, intraindividual standard deviation is another indicator that has been used to measure intraindividual variability in previous studies.^36^, ^37^ However, intraindividual standard deviation is affected by the mean; the higher the mean, the greater the intraindividual standard deviation. The CV is calculated by dividing the standard deviation by the mean. Therefore, the use of CV is appropriate for this study, in which the average time required for a series of actions in TUG decreased after CSF elimination, and the variability in gait velocity was compared before and after CSF elimination.

The present study had several limitations. First, this study was carried out at a single institute with a limited number of samples. Second, because of the lack of data on TUG‐CV from healthy controls, we could not determine whether the TUG‐CV of iNPH in this study was abnormal or not. Third, the study participants had probable iNPH, but not definite iNPH. Therefore, the results of the present study should be reinforced in the future, and further studies using larger sample sizes and normal control groups are required to better understand gait velocity variability in iNPH.

## Disclosure statement

H Kazui has received speaker's honoraria from Integra Japan. The other authors declare no conflict of interest.

## Data Availability

The data that support the findings of this study are available from the corresponding author upon reasonable request.
